# Putative malignant hyperthermia mutation Ca_V_1.1-R174W is insufficient to trigger a fulminant response to halothane or confer heat stress intolerance

**DOI:** 10.1016/j.jbc.2023.104992

**Published:** 2023-06-29

**Authors:** Wei Feng, Jose R. Lopez, Shane Antrobus, Jing Zheng, Arkady Uryash, Yao Dong, Donald Beqollari, Roger A. Bannister, Philip M. Hopkins, Kurt G. Beam, Paul D. Allen, Isaac.N. Pessah

**Affiliations:** 1 Department of Molecular Biosciences, University of California Davis, Davis, California, USA; 2 Department of Research, Mount Sinai Medical Center, Miami Beach, Florida, USA; 3 Department of Medicine-Cardiology Division, University of Colorado Anschutz Medical Campus, Aurora, Colorado, USA; 4 Institute of Medical Research at St James’s, University of Leeds, Leeds, United Kingdom; 5 Department of Physiology and Biophysics, University of Colorado Anschutz Medical Campus, Aurora, Colorado, USA

**Keywords:** malignant hyperthermia susceptibility, heat stress intolerance, voltage-activated Ca^2+^ channels, ryanodine receptor, TRPC3/6, knock-in mouse, muscle diseases

## Abstract

Malignant hyperthermia susceptibility (MHS) is an autosomal dominant pharmacogenetic disorder that manifests as a hypermetabolic state when carriers are exposed to halogenated volatile anesthetics or depolarizing muscle relaxants. In animals, heat stress intolerance is also observed. MHS is linked to over 40 variants in *RYR1* that are classified as pathogenic for diagnostic purposes. More recently, a few rare variants linked to the MHS phenotype have been reported in *CACNA1S*, which encodes the voltage-activated Ca^2+^ channel Ca_V_1.1 that conformationally couples to RyR1 in skeletal muscle. Here, we describe a knock-in mouse line that expresses one of these putative variants, Ca_V_1.1-R174W. Heterozygous (HET) and homozygous (HOM) Ca_V_1.1-R174W mice survive to adulthood without overt phenotype but fail to trigger with fulminant malignant hyperthermia when exposed to halothane or moderate heat stress. All three genotypes (WT, HET, and HOM) express similar levels of Ca_V_1.1 by quantitative PCR, Western blot, [^3^H]PN200-110 receptor binding and immobilization-resistant charge movement densities in flexor digitorum brevis fibers. Although HOM fibers have negligible Ca_V_1.1 current amplitudes, HET fibers have similar amplitudes to WT, suggesting a preferential accumulation of the Ca_V_1.1-WT protein at triad junctions in HET animals. Never-the-less both HET and HOM have slightly elevated resting free Ca^2+^ and Na^+^ measured with double barreled microelectrode in vastus lateralis that is disproportional to upregulation of transient receptor potential canonical (TRPC) 3 and TRPC6 in skeletal muscle. Ca_V_1.1-R174W and upregulation of TRPC3/6 alone are insufficient to trigger fulminant malignant hyperthermia response to halothane and/or heat stress in HET and HOM mice.

Malignant hyperthermia susceptibility (MHS) is a pharmacogenetic disorder that manifests as a hypermetabolic state when susceptible carriers are exposed to volatile anesthetics or depolarizing muscle relaxants (1). The pharmacogenetic disorder observed in humans is recapitulated in mouse models and a number of other species expressing human MHS mutations and affected mice invariably have shown intolerance to mild to moderate heat stress (2, 3, 4, 5, 6). MHS has been associated with an elevated skeletal muscle resting intracellular calcium concentration ([Ca^2+^]_i_) (4, 7) and directly related to an uncontrollable and rapid increase of intracellular Ca^2+^ upon exposure of susceptible individuals to triggering agents. This disorder has been directly linked with defects in *RYR1*, *CACNA1S*, and *STAC3* genes that encode three of five essential proteins that make up the fundamental unit for functional excitation–contraction (EC) coupling in skeletal muscle (8, 9, 10). *RYR1* encodes the intracellular Ca^2+^ release channel RyR1, a gene that is highly polymorphic with more than 1800 expressed variants of which more than 40 have been classified as pathogenic for MHS (11). *CACNA1S* encodes Ca_V_1.1, which is the α1 subunit of the skeletal muscle voltage-dependent L-type calcium channel and has been suggested to account for ∼1% of the cases associated with a positive diagnosis for MHS based on a positive result from the clinical gold standard *in vitro* contracture test (IVCT) (12). Most variants within RyR1 and Ca_V_1.1 are predicted to be either benign or are variants of unknown significance (11). To date, only six, mostly very rare, Ca_V_1.1 variants have been associated with MHS: Ca_V_1.1-R174W, -T1009K, -R1086H/C/S and T1354S (11, 13, 14, 15, 16, 17, 18, 19, 20, 21, 22).

Ca_V_1.1-R174W is within the S4 segment of repeat I, T1009 is within the S5-S6 linker of repeat III, R1086 is located in the cytoplasmic loop between repeats III and IV, and T1354 is located in the S5-S6 linker of repeat IV; all highly conserved regions. Functional studies of Ca_V_1.1-R174W and Ca_V_1.1-R1086H expressed in Ca_V_1.1-null myotubes, which lack expression of Ca_V_1.1-WT, have demonstrated increased sensitivity to caffeine-induced Ca^2+^ release (14, 16, 18, 21), while Ca_V_1.1-null myotubes expressing Ca_V_1.1-T1354S were shown to have increased action potential-evoked Ca^2+^ release in the presence of 2 mM caffeine (16).

Interestingly, unlike Ca_V_1.1-WT and Ca_V_1.1-T1354S, Ca_V_1.1-R174W supports normal EC coupling without detectable L-type Ca^2+^ current. Ca_V_1.1-R174W is at a highly conserved position in a region of the repeat I S4 helix, known to be essential for coupling depolarization to activation of L-type Ca^2+^ current *via* Ca_V_1.1 (18, 19, 23). This variant was first detected in a patient who developed a suspected malignant hyperthermia (MH) reaction under anesthesia and who was subsequently diagnosed with MHS through IVCT (15). Expressed in Ca_V_1.1-null (dysgenic) mouse myotubes, Ca_V_1.1-R174W was shown to result in significantly increased sensitivity to both caffeine and the volatile anesthetic halothane (18, 19), which are the standard agents used in the IVCT for clinical diagnosis of MHS (8, 24).

One possible weakness of the previous studies of Ca_V_1.1-R174W is that the caffeine and halothane sensitivity were assayed in myotubes derived from dysgenic myoblasts stably transfected with Ca_V_1.1-R174W. If the Ca_V_1.1-R174W were underexpressed in these myotubes relative to RyR1, the uncoupled RyR1s could have elevated Ca^2+^ leak as observed for the entirely uncoupled RyR1s in nontransfected dysgenic myotubes (25), and this elevated leak could have produced the increased sensitivity to caffeine and halothane. Furthermore, there have been to date, no animal studies testing whether any of the Ca_V_1.1 mutations suspected of conferring MHS in humans confer signs of classical fulminant MH in knock-in mice similar to what has been shown in knock-in mice expressing mutations in *RYR1*, which have been classified pathogenic for human MH after exposure to volatile anesthetics. Interestingly, these *RYR1* knock-in mice also have intolerance to mild to moderate environmental heat stress.

To directly address whether Ca_V_1.1-R174W results in an MHS phenotype in mice, we inserted the putative MHS-linked human *CACNA1S*-p.R174W mutation into the 129SV mouse genome and backcrossed the resulting knock-in mice to the C57Bl6J line. Here, we report that both heterozygous (HET) and homozygous (HOM) *Cacna1s*-p.R174W mice have normal survival and reproductivity when maintained under home cage conditions. Contrary to expectations, neither HET nor HOM mice displayed adverse outcomes characteristic of a fulminant MH syndrome when exposed to halothane anesthesia, nor are they sensitive to moderate environmental heat stress.

## Results

### CACNA1s knock-in construct, sequence verification and genotyping, and expression of Ca_V_1.1-R174W mRNAs

**Figure 1:**
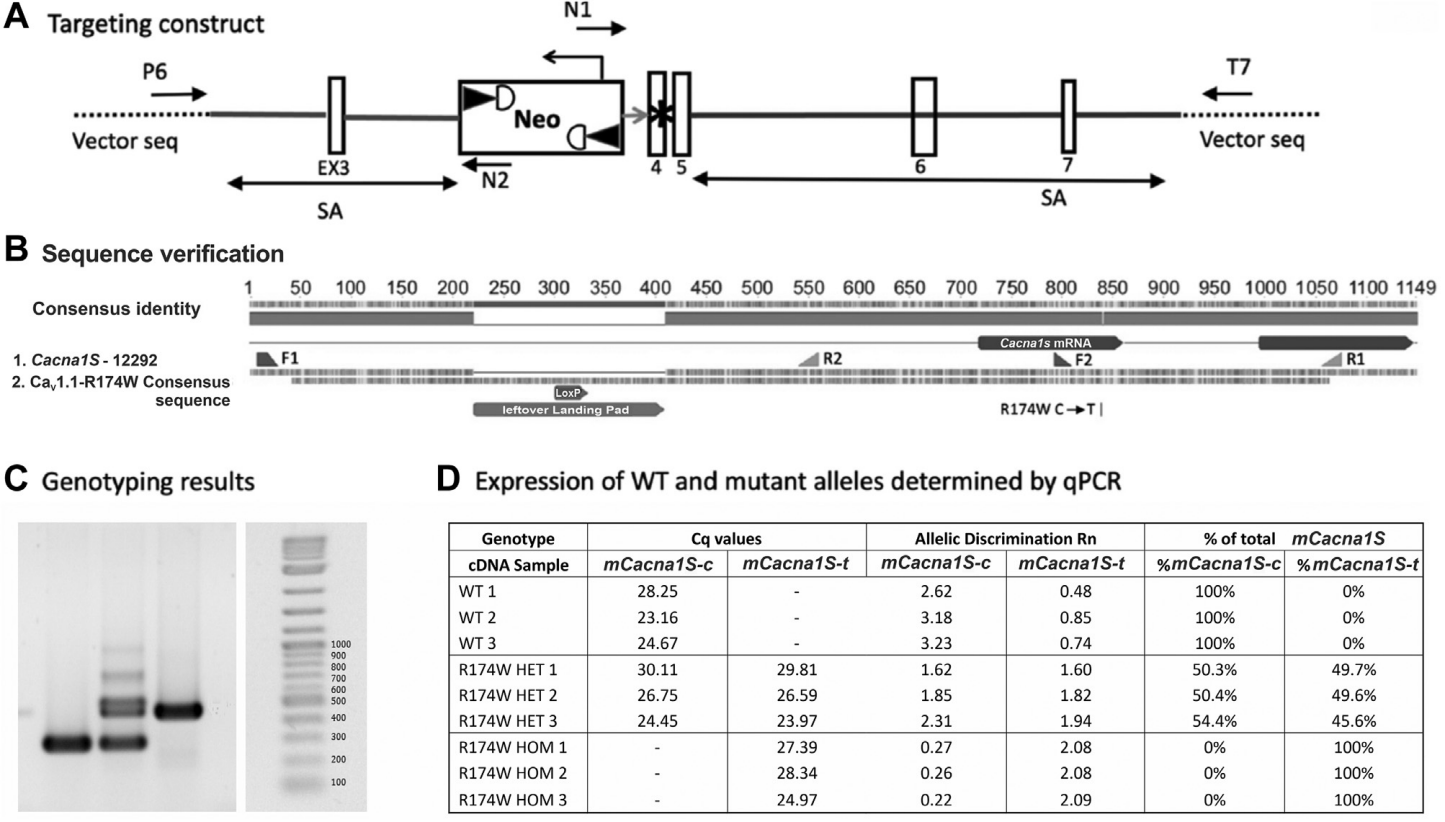
**Targeting vector,confirmationof R174W C*acna1s*DNA sequence, genotyping, and mRNA expression.***A*, targeting strategy to produce Ca_V_1.1-R174W knock in mouse line (see Experimental procedures for details). *B*, the R174W *Cacna1s* mutation was confirmed by sequencing the gene of a homozygous R174W mutant mouse in the region surrounding the R174W mutation locus and aligning it with the sequence of the *Cacna1s* gene. All animals were genotyped before and after being used for experiments. *C*, representative genotyping PCR results for homozygous WT, heterozygous WT/Ca_V_1.1-R174W, and homozygous Ca_V_1.1-R174W. *D*, quantitative real-time PCR for mouse *Cacna1s* SNP confirmed an equal expression level of *Cacna1s* mRNA in heterozygous animals.

The targeting vector used to knock-in putative MHS mutation Ca_V_1.1-R174W is shown in Figure 1*A*. Genomic DNA from a mouse HOM for the Ca_V_1.1-R174W mutation was amplified by PCR using primers F1 and R1 (Fig. 1*B*). The PCR product included the Ca_V_1.1-R174W mutation site and the intron region used for targeting during knock-in cloning. Sequencing of the PCR product was done using primers F1, F2, R1, and R2. Ca_V_1.1-R174W sequencing results aligned with the *Cacna1s* sequence and confirmed the presence of the mutation and the leftover landing pad containing the LoxP site (Fig. 1*B*). All other sequence was identical to *Cacna1s* in the database (Fig. 1*B*). Quantitative reverse transcription-PCR showed that transcripts for both Ca_V_1.1-WT and Ca_V_1.1-R176W were detected with a ratio of 1:1 in HET skeletal muscle, whereas only the respective transcripts for both Ca_V_1.1-WT and Ca_V_1.1-R176W were detected in WT and HOM samples, respectively (Fig. 1, *C* and *D*).

### Ca_V_1.1-R174W does not trigger fulminant MH in HET nor HOM mice

**Figure 2:**
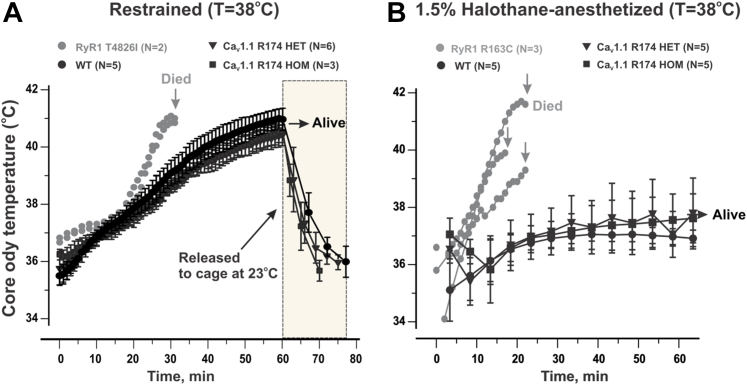
**Ca**_**V**_**1.1-R174W is not sufficient to confer intolerance to heat stress or halothane anesthesia.***A*, all mice were tested while restrained under elevated ambient temperature (38 ^°^C) as described in Experimental procedures to assess heat stress intolerance until they triggered with fulminant MH or for a maximum time of 60 min. *B*, a separate cohort of mice were induced with 2% halothane at room temperature followed by maintenance anesthesia with 1.5% halothane on a bed maintained at 37 to 38 °C for up to 60 min or until they triggered with fulminant MH. RyR1-T4826I and RyR1-R163C MHS mice served as positive controls for heat stress and halothane intolerance assays, respectively. In *panels A* and *B*, core body temperatures were monitored (1 reading/2 min) for up to 60 min. In *panel A*, temperature was monitored for up to 60 min in restraint at 38 °C and an additional 15 min (1 reading/5 min) when mice were returned to the home cage at 23 °C. All data are presented as mean ± SD from data taken from the number of mice indicated in each group. MH, malignant hyperthermia; MHS, malignant hyperthermia susceptibility.

Mice were subjected to an elevated ambient temperature (38 °C) without or with maintenance anesthesia with 1.5% halothane applied through a nose-cone for up to 60 min. Under either of these conditions, heat stress alone or heat stress + anesthesia, were sufficient to trigger fulminant MH in HET (n = 6 and n = 5, respectively) or HOM (n = 3 and n = 5, respectively) mice survived the protocol (Fig. 2, *A* and *B*). Awake HET and HOM mice survived the heat stress alone protocol lasting 60 min despite a rise in their core body temperature to ∼40.7 °C (Fig. 2*A*). Upon return to their home cages at 23 to 24 °C, both HET and HOM mice recovered to their initial core body temperatures within 20 min and failed to trigger over the ensuing 24 h when they were sacrificed. Mice subjected to 1.5% halothane maintenance anesthesia maintaining their body temperature of 37 to 38 °C, an environmental temperature empirically determined to maintain their baseline core body temperature during anesthesia, failed to exhibit hyperthermia or advance to skeletal muscle rigidity over the 60 min test period. Once returned to their home cage HET and HOM mice did not exhibit fulminant MH (Fig. 2*B*). Similar stress conditions have been shown to readily trigger fulminant events in mouse lines expressing verified *RYR1* MHS mutations, including RyR1-Y522S (3), RyR1-R163C (4), RyR1-T4826I (26), and RyR1-G2435R (6). To verify MHS under identical experimental conditions used to test Ca_V_1.1-R174W HET and HOM mice, we tested HET RyR1-R163C and HOM RyR1-T4826I mice as positive controls. In contrast to HET and HOM Ca_V_1.1-R176W, HOM RyR1-T4826I mice (n = 2) died of heat stress intolerance within 30 min of commencing the heat stress alone protocol, exhibiting a biphasic response of core body temperature with a very steep rise commencing at 20 min and reaching 41 °C at time of death (Fig. 2*A*). Death was associated with whole-body hypercontraction of skeletal muscles. When subjected to the heat stress + anesthesia protocol HET RyR1-R163C mice (n = 3) exhibited a steep rise in core body temperature that commenced soon after induction and culminated with fulminant MH between 17 and 23 min (Fig. 2*B*). Four Ca_V_1.1-R174W HET mice surviving their initial exposure to the anesthesia protocol were retested at a bed temperature of 38 to 39 °C and survived without triggering fulminant MH (data not shown).

### HOM expression of Ca_V_1.1-R174W ablates L-type Ca^2+^ currents

**Figure 3:**
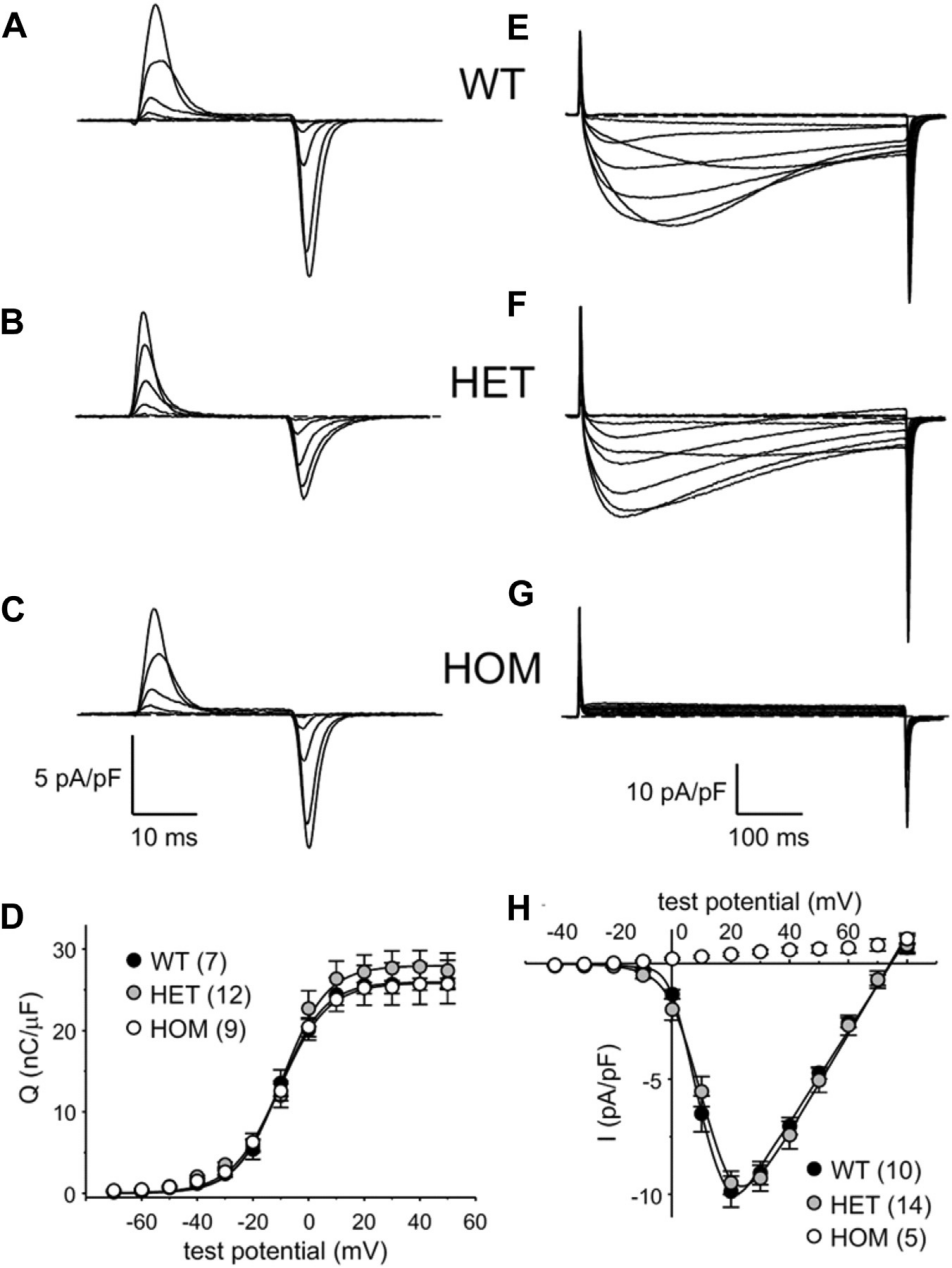
**R174W HOM FDB fibers do not conduct L-type Ca**^**2+**^**current under standard recording conditions but R174W HET fibers have current amplitudes similar to WT fibers.** Representative recordings of intramembrane charge movements elicited by 25 ms depolarizations from −80 mV to −60 mV, −40 mV, −20 mV, 0 mV, and +20 mV are shown for WT (*A*), HET (*B*), and HOM (*C*) FDB fibers. *D*, Q-V relationships corresponding to the charge movements shown in panels (*A*–*C*). Charge movements were evoked at 0.1 Hz by test potentials ranging from −70 mV through +50 mV in 10 mV increments. Representative recordings of L-type currents elicited by 500 ms step depolarizations from −50 mV to −20 mV, −10 mV, 0 mV, +10 mV, +20 mV, +30 mV, +40 mV, + 50 mV and +60 mV to +70 mV are shown for WT (*E*), HET (*F*), and HOM (*G*) fibers. *H*, peak I-V relationships corresponding to the current families shown in panels (*E* and *F*). Currents were evoked at 0.1 Hz by test potentials ranging from −40 mV through +80 mV in 10 mV increments. The smooth curves in panels (*D* and *H*) are plotted according to Equations 1 and 2, respectively, with fit parameters displayed in Table 1. The numbers of analyzed fibers are indicated in parentheses; error bars represent ± SEM. FDB, flexor digitorum brevis; HET, heterozygous; HOM, homozygous.

Similar maximal intramembrane charge movement with no detectable alterations in voltage-dependence was observed in WT, HET, and HOM flexor digitorum brevis (FDB) fibers (all parameters *p* < 0.05, ANOVA; Fig. 3, *A*–*D* and Table 1), indicating a similar number of total Ca_V_1.1 channels present in the transverse tubules of all three genotypes. Robust L-type Ca^2+^ currents were present in WT fibers (I_dens_ = −9.9 ± 0.7 pA/pF at +20 mV, *n* = 10, whereas HOM fibers displayed no inward Ca^2+^ current (*n* = 5; Fig. 3, *E*–*H* and Table 1), similar to our earlier recordings from dysgenic myotubes expressing a yellow fluorescent protein-fused Ca_V_1.1 R174W construct (18, 19). Quite unexpectedly, HET fibers had an average peak current density similar to that measured in WT fibers (I_dens_ = −9.5 ± 0.5 pA/pF at +20 mV, *n* = 14, *p* > 0.05; Fig. 3, *E*, *F* and *H* and Table 1). Statistical analysis (*t* test) of current density between those of HET and HOM indicate no significant difference (*p* > 0.05). Taken together, the nearly equivalent charge movement and current amplitudes between WT and HET fibers suggested a preferential t-tubular accumulation of the WT allele in the HET mouse.

**Table 1:** Conductance and charge movement fit parameters

Genotype	Q-V			G-V			
	Q_ma__x_	V_Q_	K_Q_	G_max_	V_1/2_	V_rev_	K_G_
	(nC/μF)	(mV)	(mV)	(nS/nF)	(mV)	(mV)	(mV)
WT	26.0 ± 7.0	−10.7 ± 4.6	6.9 ± 2.5	202 ± 39	10.1 ± 4.0	74.9 ± 3.6	4.1 ± 1.1
	(7)			(10)			
HOM	26.0 ± 2.3	−10.3 ± 4.0	8.2 ± 2.5	no current			
	(9)			(5)			
HET	28.0 ± 6.9	−9.7 ± 5.2	6.8 ± 3.5	218 ± 43	11.3 ± 5.3	74.0 ± 7.2	5.1 ± 0.9∗
	(12)			(14)			

Data are given as mean ± SD with the numbers in parentheses indicating the number of FDB fibers tested. Charge movement and conductance were fit by Equations 1 and 2|, respectively. A significant difference is indicated for the HET G-V relationship relative to WT (∗denotes *p* < 0.05 unpaired *t* test). No significant differences in the Q-V relationships were observed among the three genotypes (one-way ANOVA). All the data at 0.05 level were significantly drawn from the normally distributed population (Normality test results with Kolmogorov–Smirnov are presented in Table S1).

### Intracellular resting calcium and sodium is slightly elevated in Ca_V_1.1-R174W HET and HOM muscle fibers

**Figure 4:**
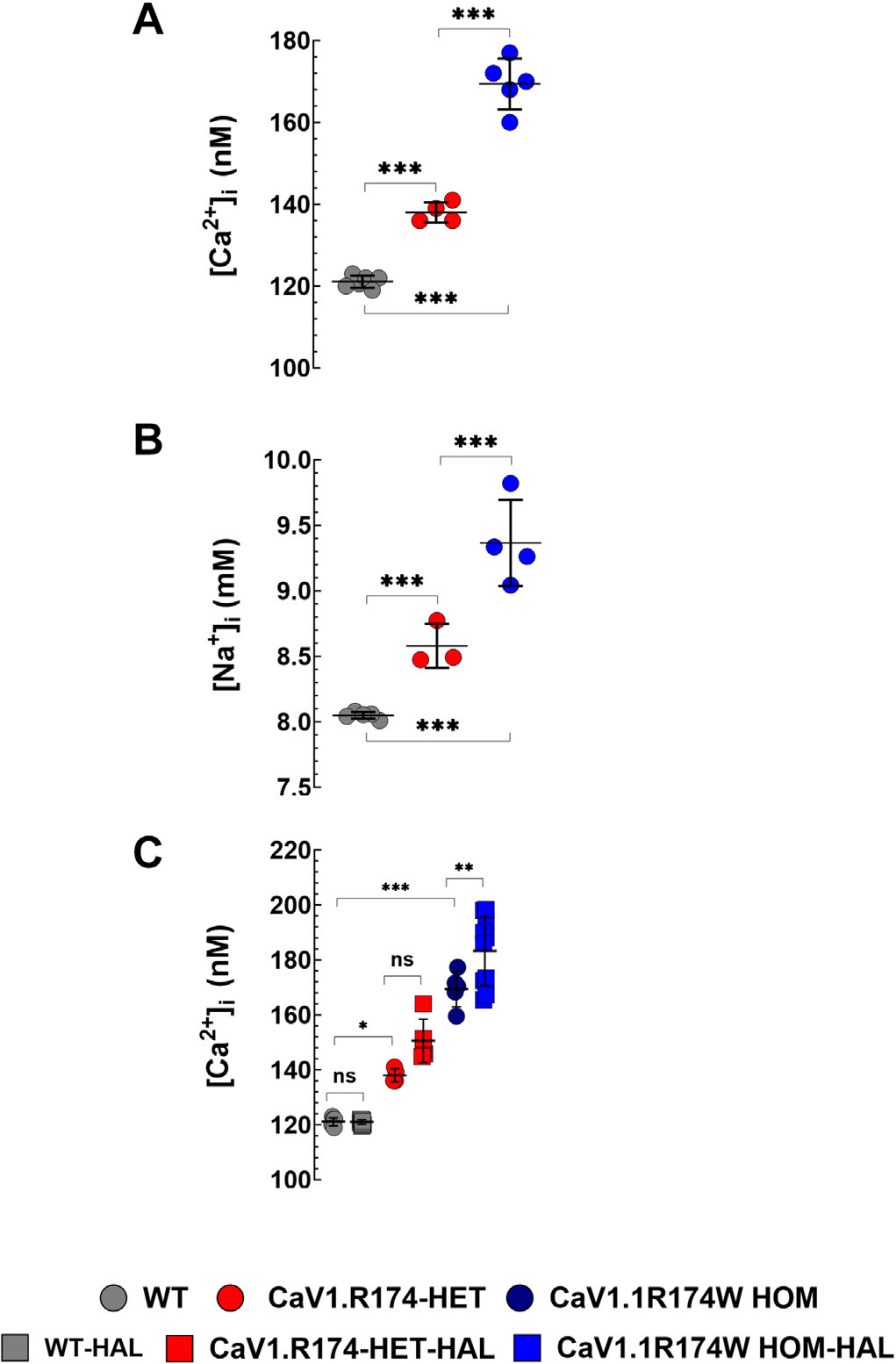
**Intracellular Ca2+ and Na2+ are modestly elevated in HET and HOM muscle.** [Ca^2+^]_i_ and [Na^+^]_i_ was measured *in vivo* on intact muscle fibers from 4-6-months WT, HET, and HOM Ca_V_1.1-R174W mice using ion-specific microelectrodes. Figure 4, *A* and *B* show that [Ca^2+^]_i_ and [Na^+^]_i_ were significantly higher in Ca_V_1.1-R174W HET and HOM than WT muscle fibers. [Ca^2+^]_i_ was 1.2-fold and 1.4-fold more elevated in Ca_V_1.1-R174W HET and HOM than WT. [Na^+^]_i_ was 1.1-fold and 1.2-fold higher in Ca_V_1.1-R174W HET and HOM than WT. Figure 4*C*, exposure to halothane 1.5% provoked a small but significant elevation of [Ca^2+^]_i_ in Ca_V_1.1-R174W muscle fibers (1.1-fold), but not in HET or WT muscle cells. Resting [Ca^2+^]_i_ measurements: WT: N_mice_= 6, n_fibers_ = 35; Ca_V_1.1-R174W HET; N_mice_ = 4, n_fibers_ = 18; Ca_V_1.1-R174W HOM: N_mice_ = 5, n_fibers_ = 35. Resting [Na^+^]_i_ measurements: WT: N_mice_= 5, n_fibers_ = 32; Ca_V_1.1-R174W HET; N_mice_ = 3, n_fibers_ = 17; Ca_V_1.1-R174W HOM: N_mice_ = 4, n_fibers_ = 20; Effects of halothane on [Ca^2+^]_i_: WT: N_mice_ = 9, n_fibers_ = 31 to 60; Ca_V_1.1-R174W HET: N_mice_ = 5, n_fibers_ = 18 to 24; Ca_V_1.1-R174W HOM: N_mice_ = 10, n_fibers_ = 55 to 60. Values are expressed as means mean ± S.D. One-way ANOVA with Tukey’s post test. ns > 0.05, ∗∗*p* < 0.01 and ∗∗∗*p* < 0.001. All the data sets passed the D'Agostino–Pearson and Shapiro–Wilk normality test (see Results and Tables S2 and S3). HET, heterozygous; HOM, homozygous.

The mean [Ca^2+^]_i_ in WT muscles was 121 ± 3 nM (N = 6 mice) compared to 138 ± 9 nM (N = 4; *p* < 0.001) in HET and 171 ± 17 nM (N = 5; *p* < 0.001) in Ca_V_1.1-R174W HOM muscles (Fig. 4*A*). We also found that [Na^+^]_i_ was elevated in muscle fibers from Ca_V_1.1-R174W (HOM > HET) than WT. The mean [Na^+^]_i_ in WT muscles was 8 ± 0.1 mM (N = 5) compared to 8.6 ± 0.4 mM (N = 3; *p* < 0.001) in HET and 9.4 ± 0.8 mM (N = 4; *p* < 0.001) in HOM Ca_V_1.1-R174W muscles (Fig. 4*B*). Exposing the animals to 1.5% halothane had no effect on [Ca^2+^]_i_ in WT (120 ± 2 nM before and 121 ± 3 nM after, N = 9; *p* = 0.99) or Ca_V_1.1-R174W HET (141 ± 9 nM before and 150 ± 20 nM after, N = 5; *p* = 0.07) skeletal muscles. Despite the fact that it did not trigger an MH episode in Ca_V_1.1-R174W HOM animals, exposure to halothane caused a significant increase in [Ca^2+^]_i_ from 167 ± 25 to 182 ± 28 (N = 10; *p* = 0.003) (Fig. 4*C*).

### Ca_V_1.1-R174W does not alter [^3^H]PN200-110 or [^3^H]Ry binding parameters

**Figure 5:**
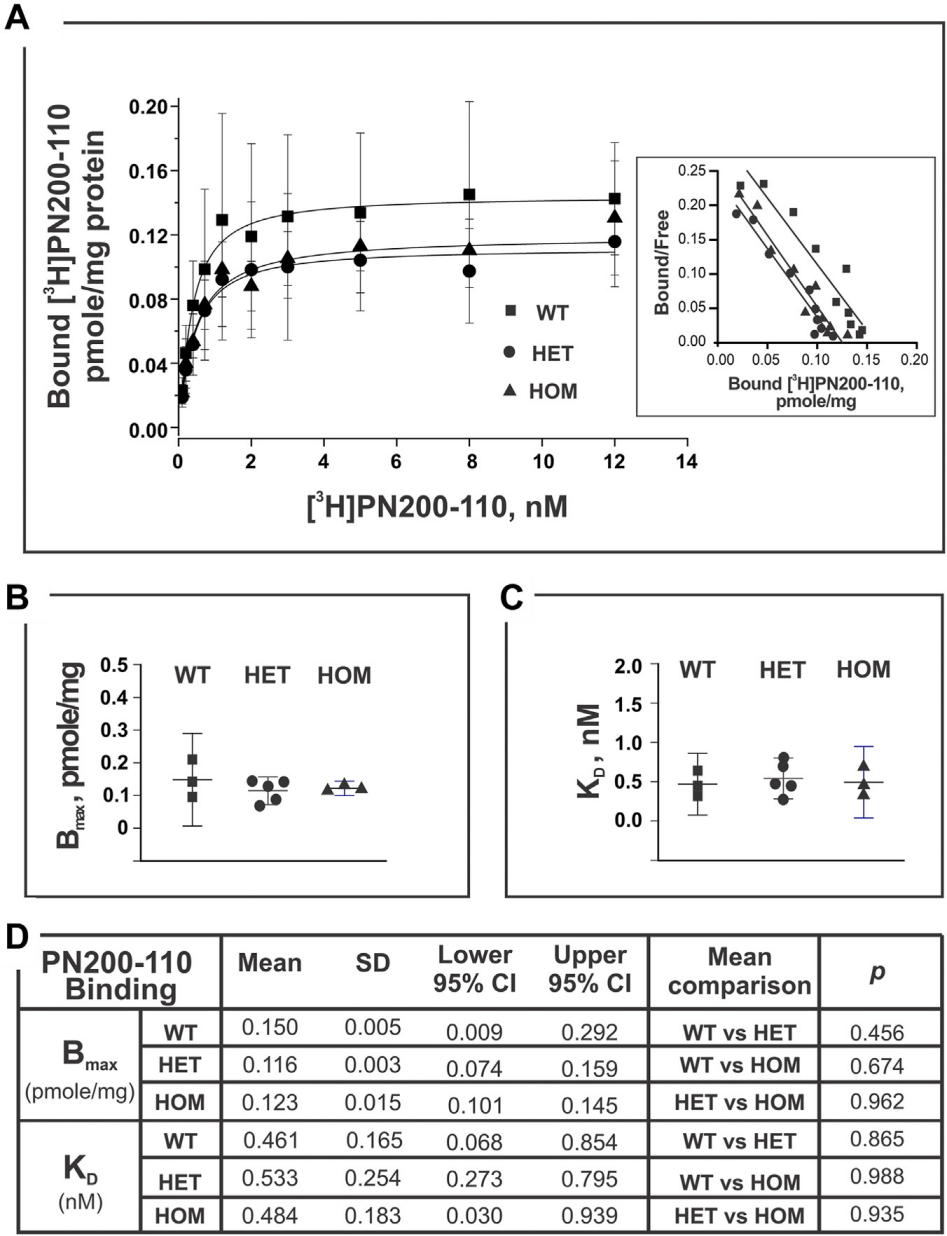
**Binding activity of [**^**3**^**H]PN200**-**110 to mouse skeletal muscle homogenate.** [^3^H]PN200-110 (0.01–12 nM; specific activity of 76.0 Ci/mmole) was used for the binding assays. Each experiment, triplicated samples at each data point, was carried out at 25 °C for 60 min in the presence of 50 μM Ca^2+^, and 500 μg/ml protein. Homogenate preparations were obtained from mice WT of N = 3; HET of N = 5, and HOM of N = 3. *Panel A* shows mean ± SD value of specific binding of the [^3^H]PN200-110. The inset is the plot of Scatchard analysis results which yield the B_max_ and *K*_D_—presented in the *panel B* and *C*. Statistical analysis (one-way ANOVA) and mean comparisons (Tukey) results are summarized in the *panel D* (using software Origin 9.1.0). Difference of the mean values of two compared groups is considered significant if the *p* < 0.05. At the level of 0.05, all population means are not significantly different based on One-Way ANOVA summary results: F = 0.16, *p* value of 0.85. HET, heterozygous; HOM, homozygous.

**Figure 6:**
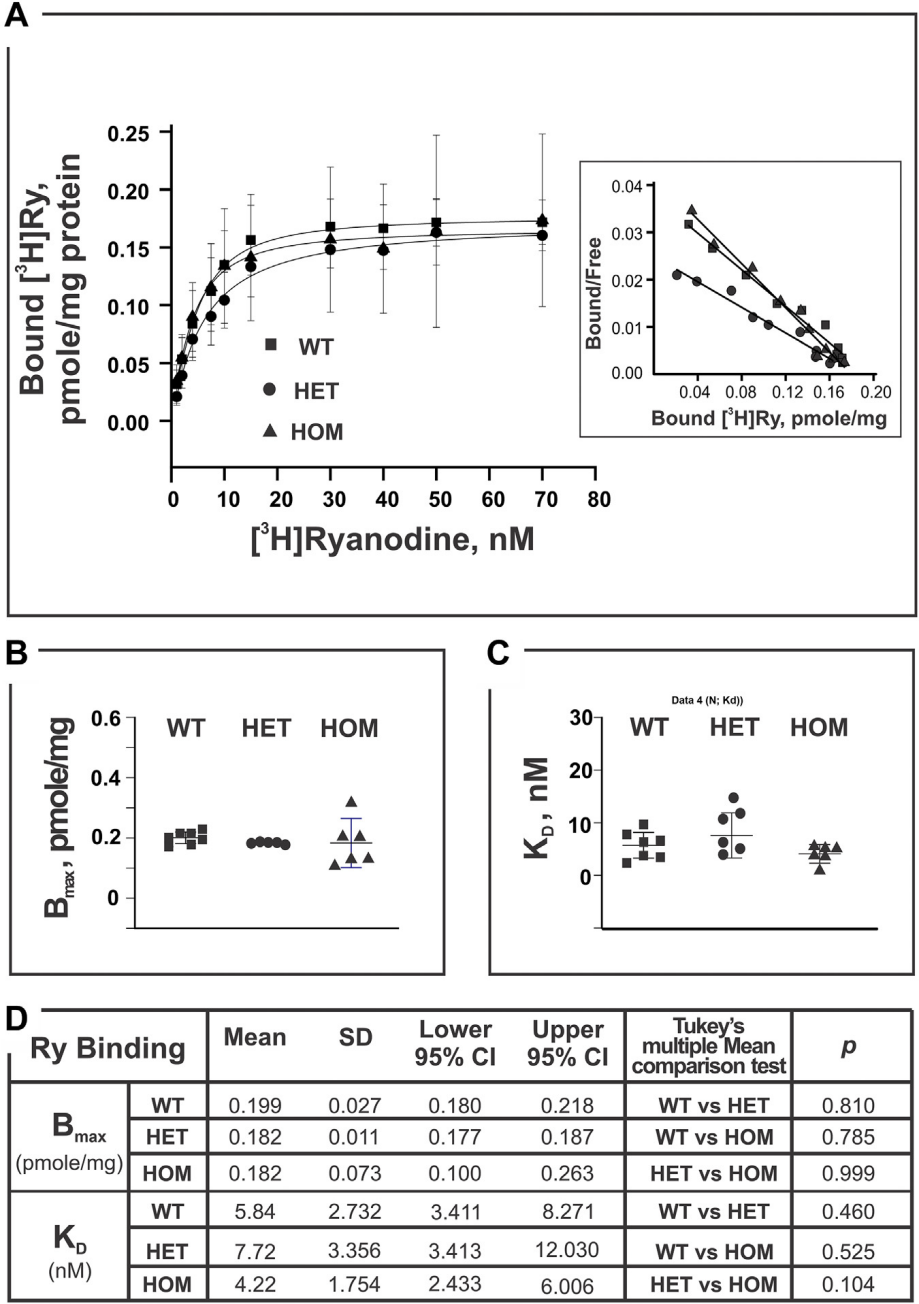
**Equilibrium high affinity binding of [**^**3**^**H]Ry to mouse skeletal muscle homogenates**. Homogenate of each mouse genotype (250 or 500 μg/ml) was incubated in buffer containing 50 μM Ca^2+^ and 1 to 70 nM [^3^H]ryanodine (specific activity 56.6 Ci/mmole) at 36 °C for 3 h. Nonspecific bindings were measured in the presence of 5 μM nonradioactive ryanodine. Each data point was mean ± SD obtained from all the independent binding experiments which had triplicated samples from the WT of N = 7, HET of N = 5, and HOM of N = 6. *Panel A* shows the specific bound [^3^H]ryanodine, the inset is the plot of Scatchard analysis from which maximal binding (B_max_) and the apparent affinity (*K*_D_) were derived. B_max_ and *K*_D_ were presented as scatter plots in *panels B* and *C*, respectively. Statistical analysis (one-way ANOVA) and mean comparisons (Tukey) results are summarized in the *panel D* (using software Origin 9.1.0). Difference of the mean values of two compared groups is considered significant if the *p* < 0.05. At the level of 0.05, all population means are not significantly different based on one-way ANOVA summary results: F = 2.57, *p* value of 0.09. HET, heterozygous; HOM, homozygous.

PN200-110 and ryanodine bind with high affinity and specificity to Ca_V_1.1 and RyR1 found in skeletal muscle and their tritiated analogs have been widely used to investigate the properties of their respective receptor binding sites. Ca_V_1.1 and RyR1 are physically and functionally coupled Ca^2+^ channels that are essential and necessary for skeletal muscle EC coupling (27). We therefore investigated whether the binding parameters for [^3^H]PN200-110 or [^3^H]Ry were altered in skeletal muscle membrane preparations harvested from the three genotypes studied. Skeletal muscle homogenates from individual WT, HET, and HOM mice were measured in parallel by titrating [^3^H]Ry or [^3^H]PN200-110 concentrations to obtain respective binding isotherms (see Experimental procedures for the details). Scatchard analyses of binding curves shown in Figure 5 indicated no significant differences in either [^3^H]PN200-110 *K*_D_ or B_max_ among the three genotypes (Fig. 5, *A*–*C*). Likewise, the [^3^H]Ry maximum binding capacity was not significantly different among genotypes (Fig. 6, *A*–*D*). However, the same preparations from HET mice indicated a trend to a higher equilibrium dissociation constant (lower affinity; *K*_D_= 7.7 nM) compared to either WT or HOM (5.8 and 4.2 nM, respectively) for [^3^H]Ry binding, though the differences did not reach statistical significance (HET *versus* WT, *p* = 0.460; HET *versus* HOM, *p* = 0.104; Fig. 6, *A*–*D*).

### Triadic protein expression profiles in WT, HET, and HOM do not differ

**Figure 7:**
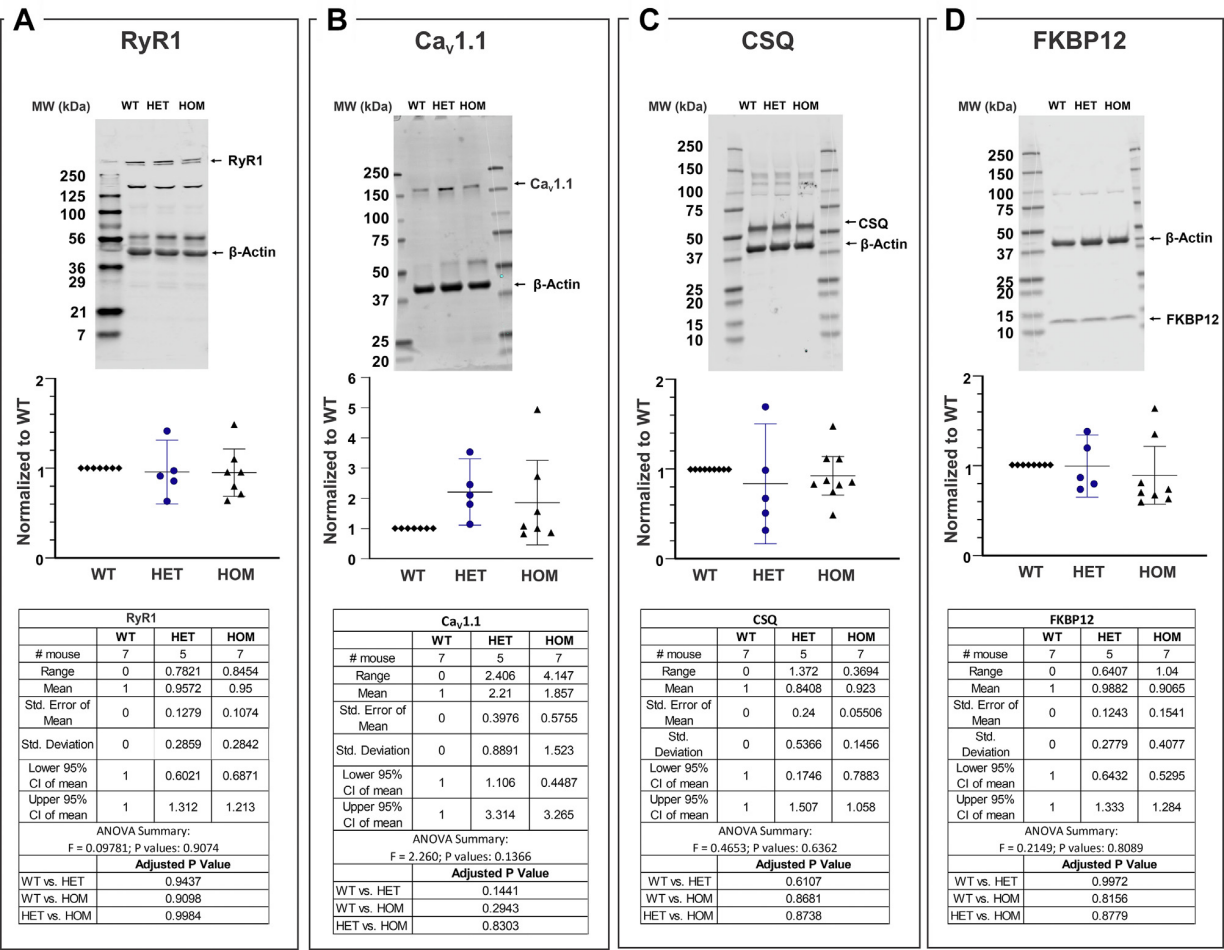
**Western blot analysis of triadic protein expression among genotypes.** The same protein preparations used for [^3^H]PN200-110 and [^3^H]Ry binding activity assessments as shown in the Figures 4 and 5 were used in the Western blot analysis. *A*, results of densitometric analysis of skeletal muscle dissected from individual mice from each of the three genotypes probed for (*A*) RyR1, (*B*) Ca_V_1.1, (*C*) calsequestrin (CSQ), or (*D*) calstabin (FKBP12). Representative Western blots used for densitometric analysis are shown as insets with GAPDH to normalize for protein loading. Densitometric values and their statistical analysis are shown in the *lower panels*. For each Western experiment, preparations from three genotypes were run on the same gel, blotted, and probed. A total of N = 7 WT, N = 5 HET and N = 7 (N = 7) mice were used for the analysis. HET and HOM signals were normalized to GAPDH or -actin, then to the WT signal on the same blot (Fig. S1). The scatter plots are the mean value with lower and upper 95% CI; SEM, SD values, the adjusted *p* values, ANOVA summary F statistic and *p* values are included in the inset tables. GraphPad Prism 9.0 was used for graph plot and statistical analysis. One-way ANOVA, Tukey’s multiple comparisons test was applied in the analysis. CI, confidence interval; CSQ, calsequestrin; HET, heterozygous; HOM, homozygous.

Preparations of skeletal muscle homogenates from individual mice (WT, N = 7; HET, N= 5; and HOM N = 7) were compared for their levels of four key triadic proteins by Western blotting; RyR1, Ca_V_1.1, calsequestrin—a high-capacity Ca^2+^-binding protein within the sarcoplasmic reticulum (SR) lumen and FKBP12 (calstabin-1), the major T cell immunophilin. Figs. 7, *A*–*D* and S1 showed that no significant differences in expression levels of these triadic proteins were detected among the three genotypes. Interestingly, the expression levels of Ca_V_1.1 in HET and HOM samples exhibited broader variability among preparations from individual animals compared to the other triadic proteins measured (Fig. 7*B*).

### TRPC3 and TRPC6 are upregulated in Ca_V_1.1-R174W skeletal muscle

**Figure 8:**
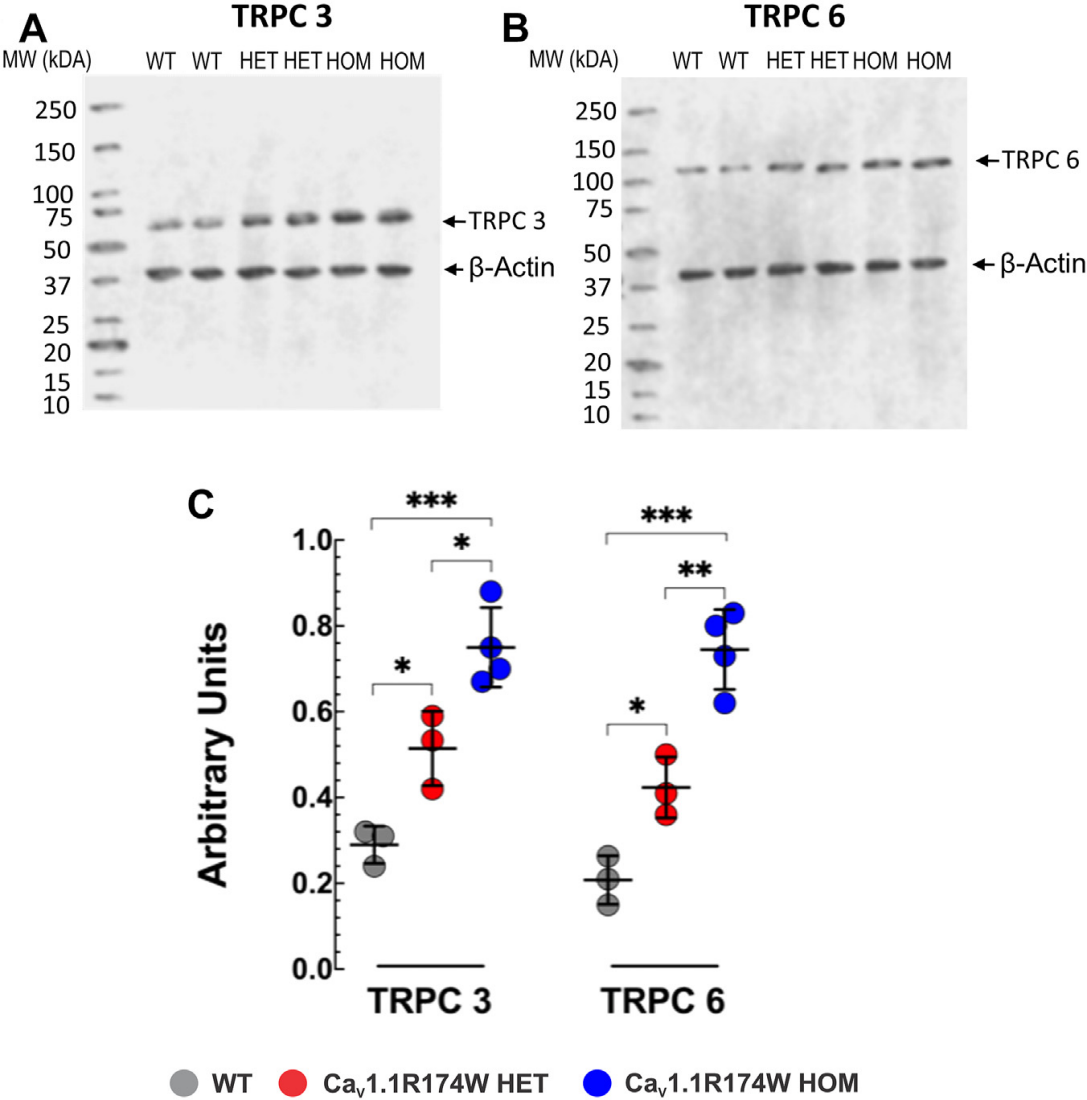
**Western blot analysis of TRPC3 and TRPC6 expression among genotypes**. Representative fluorescent Western blot analysis of the expression of TRPC3 (*panel A*) and TRPC6 (*panel B*) proteins in WT, HET, and HOM mice (*Top*Fig. 8). Densitometric analysis of individual experiment fluorescent Western blots shown in *panel C*. The proteins densitometric values are expressed as mean ± SD from three WT, three HET, and four HOM mice. ∗*p* < 0.05; ∗∗*p* < 0.01; ∗∗∗*p* < 0.001. All the data sets passed the Shapiro–Wilk normality test (Results see Table S3). HET, heterozygous; HOM, homozygous; TRPC, transient receptor potential canonical.

Recent experimental findings indicate that skeletal muscle in mice expressing pathogenic MHS RyR1 mutations exhibit altered intracellular Ca^2+^ and Na^2+^ homeostasis that can be attributed, at least in part, to dysregulation of the transient receptor potential canonical (TRPC) channels, TRPC3 and TRPC6 (28, 29, 30). Interestingly, muscle homogenates prepared from Ca_V_1.1-R174W HET or HOM mice showed significantly increased densities of both proteins compared to WT, with HOM expressing more elevated levels than HET, suggesting a gene-dose effect (Fig. 8).

## Discussion

To date, five Ca_V_1.1 variants at three distinct functional domains have been identified as potentially pathogenic for MHS based on IVCT results, R174W, T1009K, and R1086H/C/S (1, 12, 21). An additional variant, Ca_V_1.1-T1354S, was reported to produce larger action-potential-induced intracellular Ca^2+^ transients during exposure to low (2 mM) caffeine concentrations compared with CaV1.1-WT expressed in dysgenic myotubes, likely influenced by enhanced Ca2+entry (16). The prevalence of Ca_V_1.1-T1354S in control populations (https://gnomad.broadinstitute.org/variant/1-201020165-T-A?dataset=gnomad_r2_1) is, however, considered too high for it to contribute a major gene effect to MHS (31). Expression studies with Ca_V_1.1-R1086H in Ca_V_1.1 null myotubes containing WT RyR1 indicated that this mutation is sufficient to elicit a leftward shift of the caffeine concentration–effect relationship suggesting it has a functional impact on SR Ca^2+^ release dynamics consistent with an MH susceptible phenotype (14). However, whether any of these variants are indeed sufficient to trigger a fulminant MH phenotype in response to exposure to a volatile general anesthetic or to adversely respond to mild to moderate heat stress *in vivo*, which are primary phenotypes of pathogenic RyR1 MHS variants expressed in mouse models, has not been addressed. A significant finding in the present study with adult HET or HOM Ca_V_1.1-R174W mice is that the variant is insufficient to confer MHS to halothane maintenance anesthesia and it does not increase susceptibility to heat stress, conditions shown to trigger lethal fulminant events in RyR1-Y522S (3), RyR1-R163C(4) RyR1-G2435R(6) and RyR1-T4626I(5) mice. To our knowledge, the Ca_V_1.1-R174W mouse line is the first to show discordance between the MHS halothane or caffeine responses in human IVCT and the ability to elicit a fulminant MH in response to triggering anesthetic or an adverse heat stress response. In this regard, the existing MH susceptible mouse lines that express RyR1 variants pathogenic for MHS in humans have both *in vitro* and *in vivo* responses expected of an MH positive phenotype.

Before concluding that the Ca_V_1.1-R174W mouse model does not reflect the human phenotype, it is worth considering the nature of the clinical reactions in the human index cases. In Eltit *et al*, 2012 (18), we described the original index case as developing muscle rigidity in response to succinylcholine and subsequently developing hypermetabolic features during inhalation maintenance of anesthesia requiring treatment with dantrolene. The patient had marked postoperative muscle stiffness and moderate rhabdomyolysis (creatine kinase 14,500 U/L). Since then, we have found the same variant in another family diagnosed with MHS (12). In this family, the index reaction occurred in the 1970s and the only available clinical information is that the proband developed masseter muscle spasm in response to succinylcholine, had marked postoperative muscle stiffness and a raised creatine kinase (value not available). It is possible that expression of the human phenotype caused by this mutation requires administration of succinylcholine with an inhalation anesthetic.

EC coupling in skeletal muscle requires precise physical interactions between voltage-gated Ca^2+^ channels (Ca_V_1.1) in the T-Tubule and RyR1 residing in the terminal cisternae of SR (27). Their interactions engage bidirectional signaling, both orthograde activation of RyR1 and retrograde activation Ca_V_1.1 current (32). There is strong evidence for reciprocal regulation of the two channels, especially orthograde suppression of RyR1 channel activity by Ca_V_1.1 that physiologically dampen both resting Ca^2+^ in the myoplasm by controlling RyR1 mediated Ca^2+^ leak and control Ca^2+^ release properties during EC coupling (18, 33, 34). It is important to note that when both Ca_V_1.1-R174W and WT complementary DNA (cDNAs) are expressed in dysgenic FDB, immobilization-resistant charge movement and Ca^2+^ current densities are restored to levels indistinguishable from those measured in FDB expressing either WT or Ca_V_1.1-R174W cDNAs alone. Considering that expression of Ca_V_1.1-R174W alone restores immobilization-resistant charge movement in the absence of Ca^2+^ current in the dysgenic FDB model and supports skeletal muscle EC coupling in mice indicates that Ca_V_1.1-R174W is capable of inserting in the T-tubule membrane and fully supports skeletal type EC coupling *in vivo*. Moreover, results from quantitative reverse transcription-PCR demonstrate that skeletal muscle from HET mice express a 1:1 ratio of WT and Ca_V_1.1-R174W mRNAs, whereas all three genotypes express indistinguishable levels of Ca_V_1.1 protein when measured by Western botting and [^3^H]PN200-110 binding analyses. Paradoxically, Ca_V_1.1-R174W is considered a disruptive mutation since the positive charge of arginine is substituted with lipophilic tryptophan in the highly conserved S4 helix of repeat 1, a region critical for sensing membrane potential (35, 36). However, the lack of an overt phenotype in HET or HOM mice, indicates that Ca_V_1.1-R174W is insufficient to disrupt EC coupling in skeletal muscle, even when mice are exposed to the triggering anesthetic halothane or moderate heat stress, as observed with HOM mice.

MH mutations residing within *RYR1* weaken several aspects of Ca_V_1.1-RyR1 bidirectional signaling across triad junctions, including the inherent suppression of RyR1-mediated Ca^2+^ leak from SR that result in chronically elevated resting [Ca^2+^ ]_i_ and depleted SR Ca^2+^ stores (4, 18, 37, 38). The loss of negative regulation of RyR1 by Ca_V_1.1 explains the abnormally high open probabilities of RyR1 channels reconstituted in bilayer lipid membranes from R163C-RyR1 (HET mouse used in the present study) and T4826I-RyR1 HET and HOM MH susceptible mouse muscle, which lack Ca_V_1.1 and negative regulation (26, 39). MH mutations also alter L-type Ca^2+^ current *via* Ca_V_1.1 (4, 37, 40). It is plausible that Ca_V_1.1-R174W disrupts other aspects of bidirectional signaling with RyR1 in a manner that chronically elevates [Ca^2+^]_i_ and [Na^+^]_i_ in both HET and HOM mice without conferring adverse outcomes in the *in vivo* tests used in this study. However, the elevated levels of these ions and the higher levels of TRPC3 and TRPC6 found in the muscle tissues of HET mice raises important questions about the causative molecular and physiological mechanisms responsible and cannot be directly explained by disrupted Ca_V_1.1-R174W–RyR1 interactions since dysgenic myotubes injected with both equimolar cDNAs for Ca_V_1.1-WT and Ca_V_1.1-R174W cDNAs display immobilization-resistant charge movement and Ca^2+^ current properties indistinguishable from fibers expressing only Ca_V_1.1-WT. Based on these expression studies we assume that only the Ca_V_1.1-WT target to the membrane to form functional Ca^2+^ release units. Therefore, other mechanisms are likely to contribute to abnormal interactions between these triadic proteins or their binding partners the mouse model *in vivo* that are not manifested in the acute expression studies using transfected dysgenic myotubes. In this regard, such effects would be independent of the presence or absence of voltage-gated Ca^2+^ entry current. Clearly more work is needed to understand the mechanism contributing to the discordance between expression studies and those performed *in vivo* and *ex vivo* using HET and HOM skeletal muscle tissues. Nevertheless, our findings of abnormal [Ca^2+^]_i_ and [Na^+^]_i_ and TRPC isoforms in Ca_V_1.1-R174W knock-in mice is in concordance with our previous studies showing increased resting [Ca^2+^]_i_ in muscle cells in MH patients, swine, and RyR1 MHS mouse lines (4, 6, 7, 41) and with studies carried out using fluorescent Ca^2+^ indicators in human and mouse myotubes expressing RyR1 MH mutations (14, 42). However, results from the current study with HET and HOM preparations indicate that elevation of TRPC3 and TRPC6 are not primary drivers of resting [Ca^2+^]_i_ in skeletal muscle. Specifically, expression of TRPC3 is 1.8- and 2.7-fold higher and TRPC6 is 2.0 and 3.6-fold higher in HET and HOM, respectively, relative to WT. The levels of these TRPC3 and TRPC6 are greater than those previously reported for RyR1-R163C HET muscle (1.6- and 1.9-fold relative to WT, respectively). By contrast, resting [Ca^2+^]_i_ levels in Ca_V_1.1-R174W HET and HOM muscles are lower than those measured in RYR1-R163C HET or those measured in the double mutant RYR1-R163+dominant negative, both of which trigger with fulminant MH in response to halothane (28). We conclude that the upregulation of TRPC3 and TRPC6 in Ca_V_1.1-R174W skeletal muscle are not the primary drivers of resting [Ca^2+^]_i_, which are more modest than previously reported (28) and are insufficient to confer either MH susceptibility or heat stress intollerance.

Resting intracellular Ca^2+^ homeostasis in muscle cells is normally in the range of 100 to 120 nM (43) and is maintained through a coordinated function of Ca^2+^ transport mechanisms that include the Na^+^/Ca^2+^-exchanger of the plasma membrane (44), a system of high capacity, but low affinity for Ca^2+^; the plasma membrane Ca^2+^ pump or the Ca^2+^ pump of the SR (SERCA), which both have a high affinity, but a low capacity for Ca^2+^, pumping Ca^2+^ either out of the cell (plasma membrane Ca^2+^ pump) or into the SR, respectively (45). In addition, a sarcolemma Ca^2+^ influx is mediated by the TRPC channels and store-operated channels (46) and a ryanodine insensitive SR Ca^2+^ leak (33). The precise mechanisms underlying intracellular Ca^2+^ alterations in MH muscle cells have not been established yet. However, MHS has been associated with an impaired function of the SR in MH human cells (47), an increased SR Ca^2+^ passive leak *via* type 1 ryanodine receptors (RyR1) (4, 25, 48), an enhancement of the Na^+^/Ca^2+^-exchanger reverse mode (49), and an increased influx of Ca^2+^ mediated by an augmented expression of transient receptor potential (28), resulting in intracellular Ca^2+^ overload. A chronic elevated intracellular Ca^2+^ in MH muscle cells may induce Ca^2+^-activation of calpains (39), which could mediate destruction of membrane protein that allows more Ca^2+^ entry, leading to further muscle intracellular Ca^2+^ dysfunction (30).

We also found a more elevated [Na^+^]_i_ in muscle fibers from Ca_V_1.1-R174W (HOM > HET) compared to WT. This is similar to previous observations in MH muscle cells with *RyR1* mutations and is consistent with upregulation of a nonspecific cation channels (TRPCs) in the sarcolemma attempting to keep SR [Ca^2+^] normal which we see here as well. Therefore, it is plausible to suggest that the elevated intracellular [Ca^2+^] observed in Ca_V_1.1-R174W muscle cells could be the result of an increase in RyR1 leak caused by reduced negative modulation due to the mutation in Ca_V_1.1 (4, 25, 28, 50). However, the fact that the observed dysfunction of [Ca^2+^]_i_ and [Na^+^]_i_ in Ca_V_1.1-R174W muscle cells was not as marked as those observed in MH mutations residing within *RYR1* which allow us to speculate that either the RyR1 leak magnitude and/or the degree of TRPC channels expression are less manifested in this dihydropyridine receptor mutation. Further experiments are needed to establish the possible mechanisms that could contribute to the observed intracellular ionic changes.

Another intriguing observation is that despite elevated [Ca^2+^]_i_ observed in Ca_V_1.1-R174W muscle, exposure to halothane 1.5% did not further increase [Ca^2+^]_i_ in HET and produced only a marginal elevation (8%) in HOM. Furthermore, it did not trigger the expected fulminant MH episode as have been observed in RyR1 MH susceptible mice (6, 30, 51). It appears that in MH muscle cells, [Ca^2+^]_i_ must be above a particular concentration threshold to trigger the MH episode upon exposure to halogenated agents. In experiments conducted in MH susceptible swine, using the muscle relaxant azumolene to titrate [Ca^2+^]_i_, we empirically found that the intracellular Ca^2+^ threshold was ∼200 nM to trigger a fulminant MH in response to halothane (52). Thus, even after prolonged exposure to halothane (up to 60 min) MHS muscle where [Ca^2+^]_i_ is <200 nM would be insufficient to trigger the MH crisis.

## Experimental procedures

### Animals

All the procedures with animals for exposure to volatile anesthesia, terminal experiments, euthanasia, and tissue collection were conducted under protocols approved by the Institutional Animal Care and Use Committee (IACUC# 19840) at the University of California, Davis. All animals were maintained in a vivarium with constant temperature and humidity with a 12:12 light–dark cycle and provided food and water ad libitum. Mice used in the studies were adults 4 to 8 months of age, including both male and/or female WT (C57BL6/J), HOM *Ryr1*-p.T4826I; HET *Ryr1*-p.R163C; HET and HOM *Cacna1s*-p.R174W.

### Creation of knock-in mouse lines

The methods for establishing the *Ryr1*-p.T4826I (5) and *Ryr1*-p.R163C (4) mouse lines and characterization of their MHS phenotype have been previously described. The HET and HOM *CACNA1S*-p.R174W knock-in mouse lines were created for the investigators by INGenious Targeting Laboratory (www.genetargeting.com). The specifics for vector design and confirmation of homologous recombination of the mutation are found in the Supporting information (methods and figures).

### Heat stress and anesthesia testing protocols

#### Heat stress

The 4 to 8-month-old mice were placed in a restrainer (Kent Scientific HLD-MS-T) and a lubricated rectal probe with bead thermistor (Warner Instruments, TA-29) inserted rectally for the duration that the mouse was in the restraint. The temperature was monitored continuously with the probe connected to a Single Channel Temperature Controller (TC-324C; Warner Instruments) and recorded every 2 min. After 5 min at room temperature (RT) (∼24 °C), the mice were placed in a temperature-controlled chamber (38 °C; Thermo Scientific Forma Series II, Model # 1087) for a maximum of 60 min or until the mouse temperature exceeded 41 °C or the animal succumbed to the heat stress. After 60 min if the mouse survived it was removed from the heat chamber and returned to RT and observed for an additional 20 min, with the rectal temperature still being recorded every 2 min. At the end of the experiment, the mice were euthanized by cervical dislocation and decapitation.

#### Halothane anesthesia

For induction, mice were exposed to 2% halothane (Sigma-Aldrich, B4388, 2-bromo-2-chloro-1,1,1-trifluoroethane ≥ 99% pure) in a small induction chamber (EZ-177 Sure-Seal Mouse Chamber; E-Z Anesthesia). After 3 to 5 min, the anesthetized mouse, as indicated by a lack of response to gentle rocking, was removed from the small chamber and placed on a warmed platform set to 38 °C as described previously (5). Its limbs were taped in place and its head placed into a nose cone (Kent VetFlo-0305) delivering 1.5% halothane. A rectal temperature probe (Warner Instruments, TA-29) was inserted. The temperature was monitored continuously and recorded every 2 min for up to 60 min (TC-324C, Warner Instruments) or until the mouse became rigid and stopped spontaneous respiration. If the mouse survived 60 min, halothane was removed from the breathing circuit and the mouse allowed to recover and were observed in their home cage for 20 min. At the end of the experiment, the mice were euthanized by cervical dislocation and decapitation. A subset of the Ca_V_1.1-R174W HET (4 mice) surviving their initial testing were subsequently exposed to the anesthesia protocol at a bed temperature of 38 to 39 °C.

### Physiological experimental preparations

#### Measurement of intramembrane charge movements and L-type Ca^2+^ currents

Whole-cell patch-clamp experiments were performed with FDBfibers 1 to 6 h following dissociation as described (53). Patch pipettes were fabricated from borosilicate glass and had resistances of ≤ 1.0 MΩ when filled with internal solution, which consisted of (mM): 140 Cs-aspartate, 10 Cs2-EGTA, 5 MgCl_2_, and 10 Hepes, pH 7.4 with CsOH; fibers were dialyzed in the whole-cell configuration for >20 min prior to recording. For recording of L-type Ca^2+^ currents, the external solution contained (mM): 145 tetraethylammonium-methanesulfonic acid, 10 CaCl_2_, 10 Hepes, two MgSO_4_, 1 4-aminopyridine, 0.1 anthracene-9-carboxylic acid, 0.002 tetrodotoxin, pH 7.4 with tetraethylammonium-OH. 1 mM LaCl_3_ and 0.5 CdCl_2_ were added to the external recording solution to block Ca^2+^ currents during charge movement recordings. N-benzyl-p-toluensulfonamide (10–100 μM; Sigma-Aldrich) was always present in the bath solution to prevent contractions. Linear components of leak and capacitive current were corrected with -P/4 online subtraction protocols. Output filtering was at 2 to 5 kHz and digitization was either at 5 kHz (currents) or 10 kHz (charge movements). Cell capacitance (C_m_) was determined by integration of a transient from −80 mV to −70 mV using Clampex 10.3 (Molecular Devices) and was used to normalize charge movement (nC/μF) and current amplitude (pA/pF). To minimize voltage error, the time constant for decay of the whole-cell capacity transient was reduced as much as possible using the analog compensation circuit of the amplifier. Q_ON_ was then normalized to C_m_ and plotted as a function of test potential (V) and the resultant Q_ON_-V relationship was fitted according to the equation:

(1) $$Q_{ON}=Q_{\max}/\left\{1+\exp\left[\left(V_{Q}-V\right)/k_{Q}\right]\right\}\text{,}$$

where Q_max_ is the maximal Q_ON_, V_Q_ is the potential causing movement of half the maximal charge, and k_Q_ is a slope parameter. I-V curves were fitted according to the equation:

(2) $$I=G_{\max}*\;\left(V-V_{rev}\right)\;/\;\left\{1+\exp\left[-\left(V-V_{1/2}\right)\;/\;k_{G}\right]\right\}\text{,}$$

where I is the normalized current for the test potential V, V_rev_ is the reversal potential, G_max_ is the maximum Ca^2+^ channel conductance, V_1/2_ is the half-maximal activation potential and k_G_ is the slope factor.

#### Recording of intracellular calcium and sodium in muscle fibers *in vivo*

[Ca^2+^]_i_ and [Na^+^]_i_ measurements were carried out *in vivo*, on left vastus lateralis fibers in euthermic (37 °C - heating pad) and anesthetized (100 mg/kg ketamine and 5 mg/kg xylazine) WT, Ca_V_1.1-R174W HET, and Ca_V_1.1-R174W HOM mice (30). Superficial fibers of the vastus lateralis were superfused with warm Ringer’s solution (37 °C) and impaled with double-barreled Ca^2+^-selective or Na^+^-selective microelectrodes. FDB membrane and ion specific potentials were recorded *via* a high-impedance amplifier (WPI Duo 773 electrometer; WPI) (30). The membrane potential from the 3 M KCl microelectrode was subtracted electronically from the ion selective microelectrode to produce a differential Ca^2+^-specific potential or Na^+^-specific potential that represents the [Ca^2+^]_i_ or [Na^+^]_i_, respectively. All voltage signals were stored in a computer for further analysis. In a different set of experiments, [Ca^2+^]_i_ was measured before and after inhalation of 1.5% halothane in air through a nasal mask (Somno-0801 with Vetflo, Kent Scientific).

Recording of [Ca^2+^]_i_ and [Na^+^]_i_ was carried out while normal Ringer solution containing (in mM) 135 NaCl, 5 KCl, 1.8 CaCl_2_, 1 MgCl_2_, 18 NaHCO_3_, 1.5 NaH_2_PO, and 5 glucose, pH 7.3 to 7.4 was used to keep muscle fibers moist. All *in vivo* experiments were performed at 37 °C.

### Biochemical assays

#### Preparations of mouse skeletal muscle homogenates

Skeletal muscle tissue was collected immediately postmortem from individual mice ranging in age from 4 to 8 months. The harvested skeletal muscle tissue from each individual animal was processed freshly for homogenate preparation or flash-frozen with liquid nitrogen to store at −80 °C for the next scheduled preparation. The frozen skeletal muscle tissue nuggets were first ground to a fine powder in a mortar bowl filled with liquid nitrogen before being resuspended in ice-cold buffer containing 300 mM sucrose, 5 mM imidazole, and Halt Protease Inhibitor Cocktail (Thermo Fisher Scientific). The suspension (kept ice-cold) was then subjected to three sequential bursts (30 s each) of a PowerGen 700D homogenizer (Thermo Fisher Scientific) at 20,000 rpm, and then filtered through a 200 μm mesh filter (SEFAR PETEX). The filtrate was centrifuged at 110,000*g* for 60 min at 4 °C. Pellets were resuspended in 300 mM sucrose and 10 mM Hepes, pH 7.4; aliquoted into microfuge tubes (∼500 μl/tube) and stored at −80°C until used. Protein concentration was determined using the DC Protein Assay kit (Bio-Rad Laboratories).

#### Receptor binding analyses

The equilibrium binding of [^3^H]ryanodine ([^3^H]Ry) to skeletal muscle homogenate (0.25–0.50 mg/ml) was measured at 36 °C for 3 h with constant shaking in buffer consisting of 1 to 70 nM [^3^H]Ry (specific activity 56.6 Ci/mmole; PerkinElmer Life Sciences), 250 mM KCl, 20 mM Hepes, pH 7.4 and 50 μM free Ca^2+^ obtained by the addition of EGTA calculated according to the software Bound and Determined (54). Nonspecific [^3^H]Ry binding was determined in the presence of a 5 μM nonradioactive ryanodine. Bound and free ligand were separated by rapid filtration through Whatman GF/B glass fiber filters using a Brandel cell harvester (Whatman) with three washes with 5 ml of ice-cold buffer (250 mM KCl, 20 mM Hepes, 15 mM NaCl, and 50 μM Ca^2+^, pH 7.4). Retained [^3^H]Ry in filters was quantified by liquid scintillation spectrometry using a scintillation counter (Beckman model 6500). Binding analysis was performed on muscle preparations from individual animals of each genotype (biological N) performed in triplicate (technical replicates) as detailed in Figure Legends.

The equilibrium binding assays of (+)-[^3^H]PN200-110 (specific activity of 76 Ci/mmol, PerkinElmer) were performed at 25 °C for 60 min in the dark, incubated in buffer containing 140 mM NaCl, 15 mM KCl, 50 μM Ca^2+^, 20 mM Hepes, pH 7.0, and 0.25 to 0.50 mg/ml skeletal muscle homogenate. Nonspecific binding was determined in the presence of 10 μM nifedipine. The bound and free ligand was separated by rapidly washing samples through the filters with 10 X volume of cold binding buffer. Binding analysis was performed on muscle preparations from the same individual animals of each genotype (N, number of biological replicates) used for [^3^H]Ry binding analysis in triplicate (technical replicates). The number of animals used was indicated in Results and figure legends.

#### Western blot analysis

Skeletal muscle membrane preparations from individual mice were analyzed separately to measure biological variability in the level of key triadic proteins within and among genotypes. Samples were denatured in SDS-PAGE sample buffer (Bio-Rad Laboratories) containing 2.5% 2-mercaptoethanol at 60°C for 5 min. Protein (10 or15 μg/lane) was loaded onto Tris-acetate 4 to 12% or 4 to 20% acrylamide gradient SDS-PAGE gels (Invitrogen), electrophoresed at 150 V for 75 min (4 °C), and then transferred to polyvinylidene difluoride membranes at 30 V for 15 h and then at 110 V for another 1 h (4 °C). Membranes were then blocked with Odyssey blocking buffer (LI-COR Biosciences) with 0.1% Tween-20 for 1 h at RT and incubated overnight at 4 °C with primary antibodies: monoclonal RyR1(34C) 1/500 to 1/1000 (DSHB, Cat# 34C, RRID: AB_528457), monoclonal dihydropyridine receptor (M3F11) 1/200 to 1/600 (DSHB, Cat# M3F11, RRID: AB_1157868, Reno, NA), monoclonal FKBP12 1/500 to 1/1000 (FKBP12_H-5, Santa Cruz Biotechnology), CSQ 1/10,000 (Abcam, Anti-Calsequestrin 1 [EPR15227 (B)] antibody ab191564); polynoclonal GAPDH 1/1000 to 1/5000 (Millipore, Cat.# ABS16); Rabbit β-Actin Polyclonal Antibody 1/1000 (Invitrogen REF PA1-16889) in blocking buffer and then washed with Tris-*buffered* saline with 0.1% Tween-20. After washing, membranes were incubated with secondary antibodies in blocking buffer (IRDye 680 nm and 800 nm, 1/10,000, LI-COR Biosciences) for 1 h at RT, then washed again and quantified with the Odyssey Imaging System (LI-COR Biosciences).

Gastrocnemius muscles from all genotypes were dissected, homogenized, and processed using total protein extraction kit (Millipore). Total protein concentration was determined using the bicinchoninic acid method (Thermo Fisher Scientific). Denatured, SDS-gel separated and membrane immobilized proteins were incubated overnight at 4 °C with primary antibodies: anti-TRPC3, dilution 1:2500 (ab51560; Abcam), anti-TRPC6, dilution 1:2500 (ab62461, Abcam), anti-actin, dilution of 1:5000 (SC8432), and secondary fluorescent antibodies (Abcam). The resolved bands were detected with a Storm 860 Imaging System (GE HealthCare Bio-Sciences). Protein levels were quantified using myImage. Analysis software (Thermo Fisher Scientific) and normalized to -actin.

### Statistical analysis

No specific power calculation was conducted prior to experimentation; sample sizes used were based on our previous studies using other MH and non-MH animal models. Randomization methods were not used to assign the animals to be studied, and blinding of the investigators was not used.

[Ca^2+^]_i_ and [Na^+^]_i_ data are presented as mean ± SD. We used histograms and the D'Agostino–Pearson test to assess the distribution of the data (Supporting information). Statistical analyses were made by one-way ANOVA with Tukey’s post test for multiple measurements with *p* < 0.05 considered significant (GraphPad Software,9.0 Inc.; https://www.graphpad.com). Data were discarded: (i) from the muscle cell whose membrane potential was less negative than minus eighty mV; (ii) when a drift of more than 5 mV between the first and the second microelectrode calibration curves in the range between pCa6–7 for Ca^2+^ selective-microelectrodes and 1 to 10 mM for the Na^+^ selective-microelectrode; or (iii) when broken microelectrode tips which did not allow us to carry out the post measurement calibration curves.

For electrophysiological and fluorescence experiments all whole-cell data are presented as mean ± SEM, or mean ± SD. Figures were made using SigmaPlot (version 11.0, SSPS Inc). Statistical comparisons were made by unpaired *t* test with *p* < 0.05 considered significant All the data at 0.05 level were significantly drawn from the normally distributed popluation (Normality test results with Kolmogorov–Smirnov are presented in Table S1. (OriginPro 2018b).

[^3^H]ryanodine and [^3^H]PN200-110 binding isotherms obtained from individual animals were fitted by nonlinear curve fitting using OriginPro 2018b. Statistical analysis for differences in ryanodine and PN200-110 binding constants (*K*_D_ and B_max_) among the three genotypes were assessed using Prism GraphPad Software to obtain mean, SD, SEM, and lower/upper 95% confidence interval of mean using one-way ANOVA with Tukey’s multiple mean comparisons test.

Analysis of Western blots was performed on the same sample membrane preparations measured for radioligand binding indicated above. HET and HOM signals were normalized to GAPDH or -actin, then to the WT signal on the same blot. Normalized band densities in HET and HOM were subsequently normalized to WT bands to generate scatter plots of mean normalized densities ± SD and lower and upper 95% confidence interval. Statistical comparisons among genotypes were performed using a one-way ANOVA with Tukey’s multiple comparisons test applied to obtain adjusted *p* values (GraphPad Prism 9.0). Summary data of F statistic and *p* values are included in Figure 7 (table inserts).

## Data availability

All data reported in this manuscript are available in the figures, Experimental procedures section, and Supporting Information section.

## Supporting information

This article contains supporting information.

## Conflict of interest

This work was prepared while R. A. B. was employed at the University of Colorado Anschutz Medical Campus. The authors declare that they have no conflicts of interest with the contents of this article.
