# Supplementary material for: Putative malignant hyperthermia mutation CaV1.1-R174W is insufficient to trigger a fulminant response to halothane or confer heat stress intolerance
Source: J Biol Chem. 2023 Jun 29;299(8):104992. doi: 10.1016/j.jbc.2023.104992 (PMC10413282; doi:10.1016/j.jbc.2023.104992)
Supplement: Supporting information [file mmc1.docx]

**SUPPORTING INFORMATION**

Putative malignant hyperthermia mutation Ca_V_1.1-R174W is insufficient to trigger a fulminant response to halothane or confer heat stress intolerance

Wei Feng^1^, Jose R. Lopez^1,2^, Shane Antrobus^1^, Jing Zheng^1^, Arkady Uryash^2^, Yao Dong^1^, Donald Beqollari^3^, Roger A. Bannister^3*^, Philip M. Hopkins^4^, Kurt G. Beam^5^, Paul D. Allen^1,4^, and Isaac. N. Pessah^1^

^1^Department of Molecular Biosciences, University of California Davis,

Davis, CA 95616, USA.

^2^Department of Research, Mount Sinai Medical Center,

Miami Beach, FL 33140, USA.

^3^Department of Medicine-Cardiology Division, University of Colorado Anschutz Medical Campus, Aurora, CO 80045, USA

^4^Institute of Medical Research at St James’s, University of Leeds,

Leeds, LS9 7TF United Kingdom.

^5^Department of Physiology and Biophysics, University of Colorado Anschutz Medical Campus, Aurora, CO 80045, USA

Correspondence:

Isaac N. Pessah

[inpessah@ucdavis.edu](mailto:inpessah@ucdavis.edu)

530-304-1439

*Current address: National Institutes of Health, Center for Scientific Review, Bethesda, MD, USA 20892. This work was prepared while R.A.B. was employed at the University of Colorado Anschutz Medical Campus. The opinions expressed in this article are the author's own and do not reflect the view of the National Institutes of Health, the Department of Health and Human Services or the United States government.

**METHODS**

***CACNA1S*-p.R174W *vector design outline*:** An 8.62kb region used to construct the targeting vector was first sub cloned from a positively identified C57BL/6 BAC clone (RP23: 178F5). The region was designed such that the long homology arm (LA) extends 5.56kb 3’ to the point mutation in exon 4 and the LoxP/FRT flanked Neo cassette is inserted 430 bp 5’ to the C🡪T point mutation engineered into exon 4. The short homology arm (SA) extends 2.63kb 5’ to the LoxP-FRT flanked Neo cassette. The targeting vector was constructed using Red/ET recombineering technology. The BAC was sub cloned into a ~2.4kb backbone vector (pSP72, Promega) containing an ampicillin selection cassette for retransformation of the construct prior to electroporation. A pGK-gb2 LoxP-FRT Neo cassette was inserted into the gene and then the mutation was engineered by overlap extension PCR. Two primary PCR fragments were generated that overlap just 5’ to the site of the point mutation. The C 🡪 T mutation was engineered into both primers. The two primary products were then mixed and used as a template in a secondary PCR reaction in which the entire sequence containing the point mutation was amplified. A 5’ endogenous restriction site, EcoRI, was used with a 3’ endogenous restriction site, EcoRI, for insertion of the secondary PCR fragment into the BAC sub-clone containing the Neo cassette. The PCR amplified region was then sequenced. The total size of the targeting construct (including vector backbone and Neo cassette) was 12.72 Kb.

The targeting construct was linearized using Asc I prior to electroporation into iTL BA1 (C57BL/6N x 129/SvEv) hybrid embryonic stem cells and clones were screened for incorporation of both targeting vector arms. Targeted embryonic stem cells were then microinjected into C57BL/6 blastocysts. Resulting chimeras with a high percentage agouti coat color were mated to wild-type C57BL/6J mice to generate F1 heterozygous offspring. Tail DNA was analyzed as described below from pups with agouti or black coat color. Targeted mice were then backcrossed with 129-*Alpl^tm1(cre)Nagy^*/J mice (Jackson Laboratories, Bar Harbor, ME) that expresses Cre recombinase in primordial germ cells to remove the Neo cassette from the targeting construct, and then backcrossed to C57BL/6J mice to first remove the Cre transgene and then to make them congenic with the WT C57BL/6J strain (Jackson Laboratories, Bar Harbor, ME) that were used as controls. Here forth, the line is referred to as Ca_V_1.1-R174W.

***Genotyping Ca_V_1.1-R174W mice*:** PCR used to determine the genotype of mice were conducted in a volume of 25 μl containing 100 ng of genomic DNA, 1 × Expand High Fidelity PCR buffer with MgCl_2_ (Roche), 200 μM dNTP (Roche), 1.3 U Expand High Fidelity enzyme mix (Roche), 500 nM upstream primer (5’-AGCATCATGGTTTCACAGAGTG-3’), 500 nM downstream primer (5’-GTTTCTGAGGCCAAGTTCAGC-3’), and sterile double-distilled water (Applied Biosystems). PCR amplification was done using the following program: 94°C for 5 min; 35 cycles of 94°C for 30 s, 59°C for 30 s, 72°C for 30 s; and final extension at 72°C for 7 min. 12.5 microliters of the PCR reactions were then run on a 1% agarose gel for visualization. Expected wild type and mutant PCR product sizes were 365 bp and 555 bp respectively.

***Quantitative Real-time PCR systems*:** For mouse CACNA1S SNP analysis, two primers and two internal hydrolysis fluorescent labeled probe (5´ end, reporter dye FAM (6-carboxyflourescein) or VIC (2′-chloro-7′phenyl-1,4-dichloro-6-carboxy-fluorescein), 3´ end, quencher NFQMGB (Non-Fluorescent Quencher Minor Grove Binding)) was designed using Primer Express software (Thermo Fisher Scientific, Carlsbad, CA. The forward primer is mCACNA1S -481f GTGAAGGCCCTTCGAGCATA, the reverse primer is mCACNA1S -567r GATGGAGTTGAGGACCACCTG, and the two probes are mCACNA1S–517C FAM-TGCGGCTGGTGTC-MGB and mCACNA1S–517T VIC-CTGTGGCTGGTGTC-MGB. Basic Local Alignment Search Tool (BLAST) of each amplicon confirmed target gene.

qPCR systems were validated using 5-fold dilutions of cDNA testing positive for the target genes. The dilutions were analyzed in triplicate and a standard curve plotted against the dilutions. The slope of the standard curve was used to calculate amplification efficiencies using the formula E = 10 ^1/-s^-1. To pass validation, all efficiencies must be greater than 90%. Each SNP assay was validated with its unique probe and the final validation was done with both probes in the same well.

***BioSprint Sample collection and automated nucleic acid preparation*:** Tissue samples were collected in RNA later and stored at -20°C. RNA later was removed and 600μl of ATL and 60μl Proteinase K was added to each well with the tissue and two grinding beads (4 mm diameter, stainless steel beads, SpexCertiprep, Metuchen, NJ) were added and the tissues homogenized in a GenoGrinder2000 (SpexCertiprep) for 2 min at 1000 strokes per minute. Total nucleic acid was extracted from the tissue lysates using a QIAcube HT a semi-automated nucleic acid workstation (Qiagen, Valencia, CA) according to the manufacturer’s instructions for the QIAamp 96 DNA QIAcube HT Kit (Qiagen).

***RT-reaction and qPCR:*** The Quantitect Reverse transcription kit (Qiagen) was used for cDNA synthesis following the manufactures directions with the following modifications. Ten microliters of RNA were digested with 1μl of gDNA WipeOut Buffer by incubation at 42°C for ten minutes and then briefly centrifuged. Then 0.5μl of Quantitect Reverse Transcriptase, 2μl Quantitect RT buffer, 0.5μl RT Primer Mix, 0.5μl 20 pmol Random Primers (Invitrogen) were added and brought up to a final volume of 20μl and incubated at 42°C for 40 minutes. The samples were inactivated at 95°C for 3 minutes, chilled, and 80μl of water was added.

Each qPCR reaction contained 20x primer and probes for the SNP assay with a final concentration of 800 nM for each primer and 80 nM for each probe and commercially available PCR mastermix (TaqMan Universal PCR Mastermix, Applied Biosystems) containing 10 mM Tris-HCl (pH 8.3), 50 mM KCl, 5 mM MgCl_2_, 2.5 mM deoxynucleotide triphosphates, 0.625 U AmpliTaq Gold DNA polymerase per reaction, 0.25 U AmpErase UNG per reaction and 5μl of the diluted cDNA sample in a final volume of 12μl. The samples were placed in 384 well plates and amplified in an automated fluorometer (ABI PRISM 7900 HTA FAST, ABI). ABI’s standard amplification conditions were used: 2 min at 50°C, 10 min at 95°C, 40 cycles of 15 s at 95°C and 60 s at 60°C. Fluorescent signals were collected during the annealing temperature and CT values extracted with a threshold of .1 and baseline values of 3-10. Before and after the qPCR run the Allelic Discrimination protocol was run to determine the normalized reporter for each SNP.

***Sequence verification of Ca_V_1.1-R174W region*:** A PCR reaction encompassing the R174W mutation, and the landing pad leftover from knock-in mouse design, was performed to confirm the presence of the Ca_V_1.1-R174W mutation. The PCR was conducted in a volume of 50 μl containing 200 ng of genomic DNA, 1 × Expand High Fidelity PCR buffer with MgCl_2_ (Roche), 200 μM dNTP (Roche), 2.6 U Expand High Fidelity enzyme mix (Roche), 500 nM upstream primer (5’-AGCATCATGGTTTCACAGAGTG-3’), 500 nM downstream primer (5’- ATGACCATGAAGAGGACGAG-3’), and sterile double-distilled water (Applied Biosystems). PCR amplification was done using the following program: 94°C for 5 min; 35 cycles of 94°C for 45 s, 59°C for 600 s, 72°C for 90 s; and final extension at 72°C for 7 min. PCR reactions were then run on a 1% agarose gel for visualization and excision. The expected band size of 1070 bp was excised and extracted using the QIAguick Gel Extraction Kit (Qaigen) according to their instructions. The excised PCR product was then sequenced (UC Davis Sequencing Core) using two forward (5’-AGCATCATGGTTTCACAGAGTG-3’, 5’-GCTTGGATGTGAAGGCCC-3’) and two reverse primers (5’-GTTTCTGAGGCCAAGTTCAGC-3’, 5’-ATGACCATGAAGAGGACGAG-3’).

**RESULTS**

**Normality testing for resting [Ca^2+^]_i_ and [Na^+^]_i_ data presented in Figure 4.**

**1.  Resting intracellular Ca^2+^:**

WT 121±3 nM

HET: 138±9 nM (p<0.001 compared to WT))

HOM: 171±17 nM (p<0.001 compared to WT)

Values expressed as mean ± SD

***D’Agostino & Pearson Test***

WT: *p value*: 0.33

HET: *p value*: 0.38

HOM: *p value*: 0.82

**2. Resting intracellular Na^+^:**

WT 8±0.1 mM

HET: 8.6±0.4 mM (p<0.001 compared to WT))

HOM: 9.4±0.8 mM (p<0.001 compared to WT)

Values expressed as mean ± SD

***D’Agostino & Pearson Test***

WT: *p value*: 0.51

HET: *p value*: 0.23

HOM: *p value* 0.63

**3. Effect of Halothane on resting intracellular Ca^2+^:**

WT: 120±2 nM (before HAL) and 121±3 (after HAL) (*p*=0.99 compared with untreated)

Values expressed as mean ± SD

***D’Agostino & Pearson Test***

WT: *p value*: 0.23 (before)

WT: *p value*: 0.28 (after)

HET: 141±9 nM (before HAL( and 150±20 nM (after HAL) (*p*=0.07 compared with untreated)

Values expressed as mean ± SD

***D’Agostino & Pearson Test***

HET: *p value*: 0.079 (before)

HET: *p value*: 0.18 (after

HOM: 167±25 nM (before HAL) 182±28 (after HAL) (*p*=0.001 compared with untreated)

Values expressed as mean ± SD

***D’Agostino & Pearson Test***

HOM: *p value*: 0.099 (before)

HOM: *p value*: 0.072 (after

**Supporting Table 1. Normality tests for conductance and charge movement fit parameters presented in Table 1 - Test of the distribution of population means:**

**Supporting Table 2. Shapiro-Wilk normality tests for intracellular Ca^2+^ and Na^+^ measured *in vivo* on intact mouse muscle fibers.**

**Supporting Table 3 Shapiro-Wilk normality tests for population of means of expressed TRPC3 and TRPC6 measured by Western blot analysis.**

| **Genotypes** | **N (animals)** | **p values** | **Passed Normality Test** |
| --- | --- | --- | --- |
| **WT** | 3 | 0.219 | Yes |
| **R174W HET TRPC3** | 3 | 0.637 | Yes |
| **R174W HOM TRPC6** | 4 | 0.446 | Yes |
| **WT** | 3 | 0.934 | Yes |
| **R174W HET TRPC3** | 3 | 0.687 | Yes |
| **R174W HOM TRPC6** | 4 | 0.606 | Yes |

**Supporting Figure 1.** For each western blot experiment summarized in Figure 7, skeletal muscle preparations from at least 3 separate animals from each of the three genotypes were run on the same gel, blotted and probed (refer to Fig.7 for summary data).

**
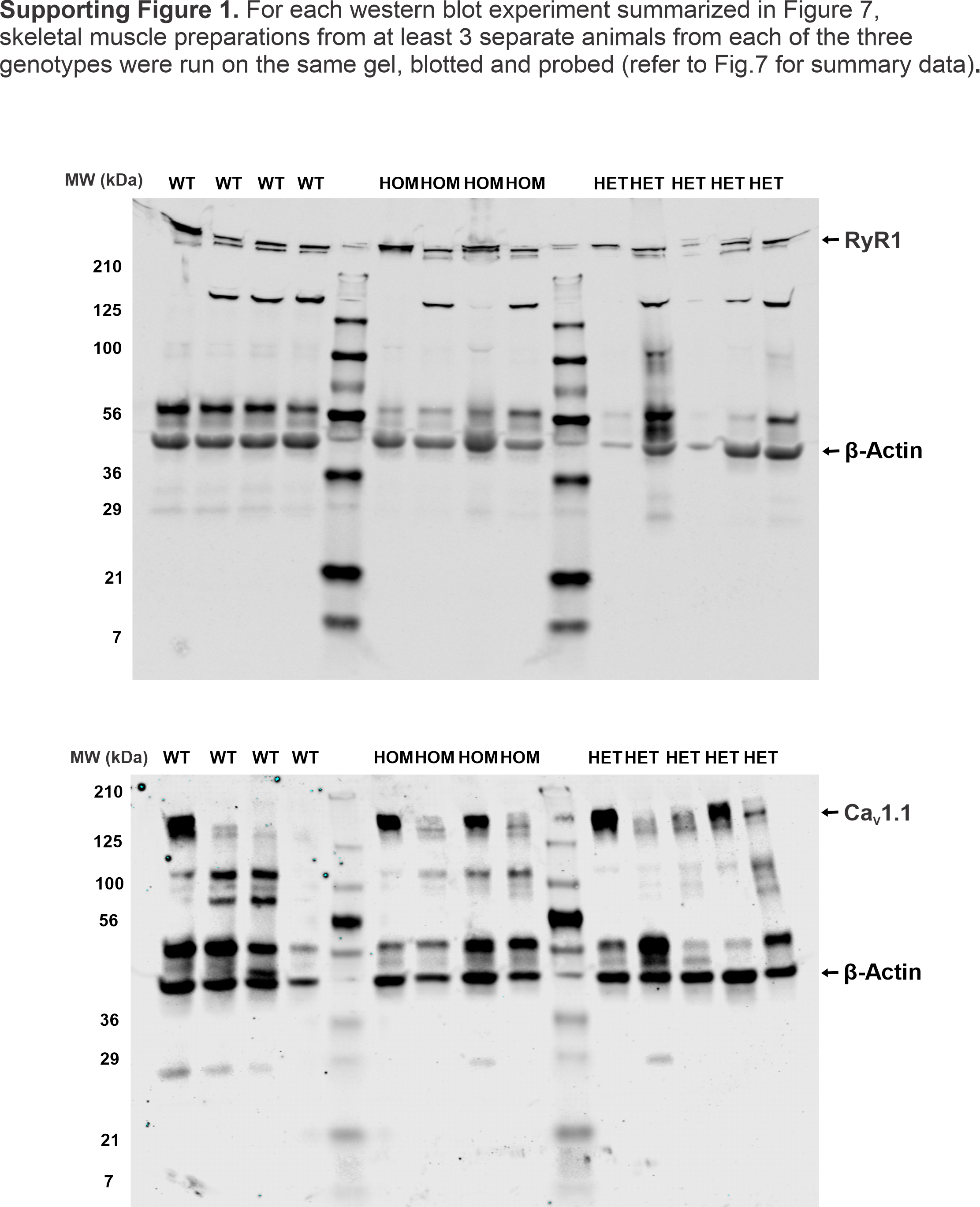
**

**
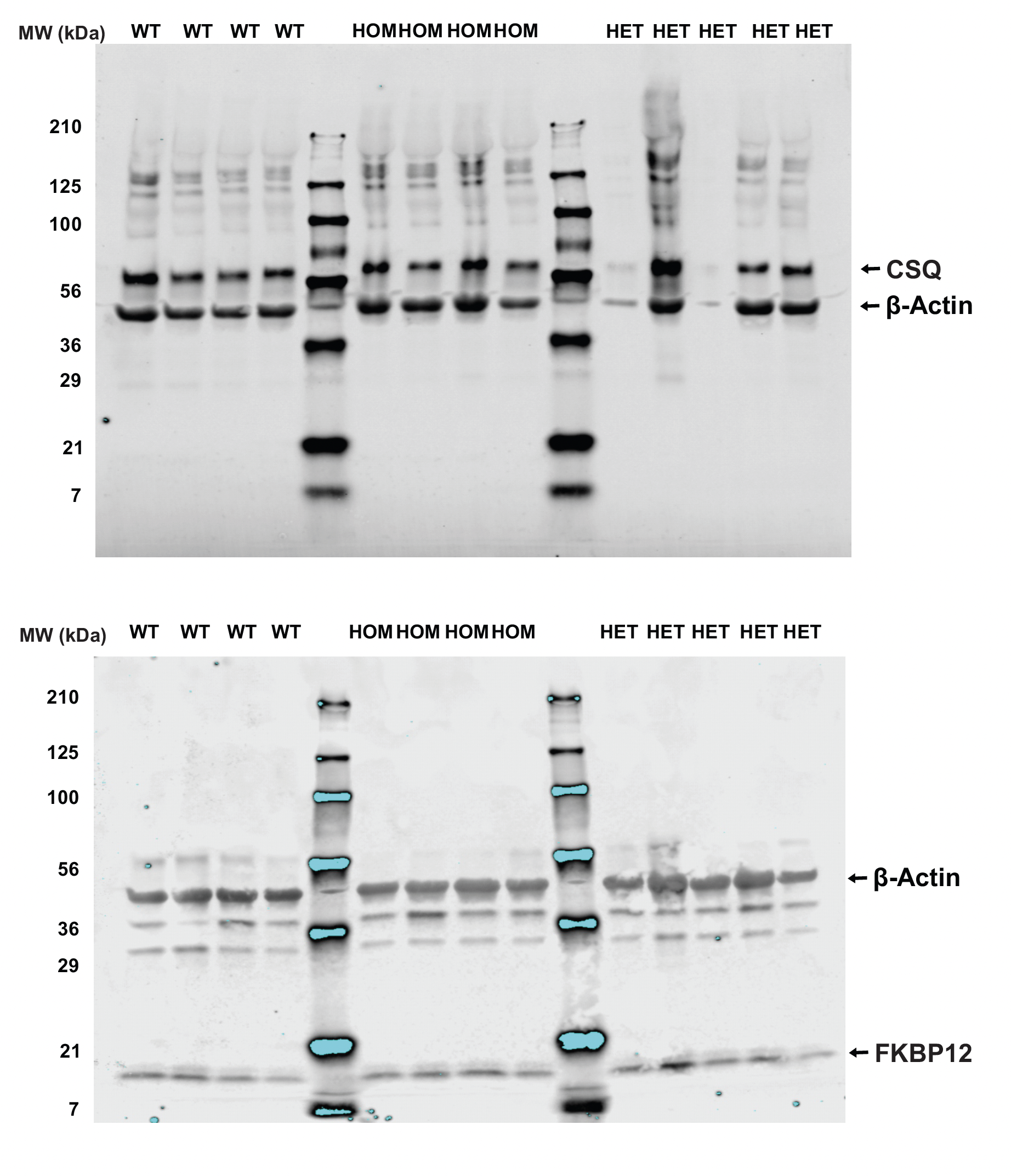
**
